# Upregulation of the chromatin remodeler HELLS is mediated by YAP1 in Sonic Hedgehog Medulloblastoma

**DOI:** 10.1038/s41598-019-50088-1

**Published:** 2019-09-20

**Authors:** M. Hope Robinson, Victor Maximov, Shoeb Lallani, Hamza Farooq, Michael D. Taylor, Renee D. Read, Anna Marie Kenney

**Affiliations:** 1 Department of Pediatric Oncology, Emory University, Atlanta, GA 30322 USA; 2 Cancer Biology Graduate Program, Winship Cancer Institute, Emory University, Atlanta, GA USA; 3 Department of Pharmacology, Emory University, Atlanta, GA 30322 USA; 4 Winship Cancer Institute, Atlanta, GA 30322 USA; 5 Developmental and Stem Cell Biology Program, The Hospital for Sick Children, Toronto, ON Canada; 6 The Arthur and Sonia Labatt Brain Tumour Research Centre, The Hospital for Sick Children, Toronto, ON Canada; 7 Division of Neurosurgery, The Hospital for Sick Children, Toronto, ON Canada; 8 Department of Surgery, Department of Laboratory Medicine and Pathobiology, and Department of Medical Biophysics, University of Toronto, Toronto, ON Canada

**Keywords:** Paediatric cancer, Developmental neurogenesis

## Abstract

Medulloblastoma is a malignant pediatric tumor that arises from neural progenitors in the cerebellum. Despite a five-year survival rate of ~70%, nearly all patients incur adverse side effects from current treatment strategies that drastically impact quality of life. Roughly one-third of medulloblastoma are driven by aberrant activation of the Sonic Hedgehog (SHH) signaling pathway. However, the scarcity of genetic mutations in medulloblastoma has led to investigation of other mechanisms contributing to cancer pathogenicity including epigenetic regulation of gene expression. Here, we show that Helicase, Lymphoid Specific (HELLS), a chromatin remodeler with epigenetic functions including DNA methylation and histone modification, is induced by Sonic Hedgehog (SHH) in SHH-dependent cerebellar progenitor cells and the developing murine cerebella. HELLS is also up-regulated in mouse and human SHH medulloblastoma. Others have shown that HELLS activity generally results in a repressive chromatin state. Our results demonstrate that increased expression of HELLS in our experimental systems is regulated by the oncogenic transcriptional regulator YAP1 downstream of Smoothened, the positive transducer of SHH signaling. Elucidation of HELLS as one of the downstream effectors of the SHH pathway may lead to novel targets for precision therapeutics with the promise of better outcomes for SHH medulloblastoma patients.

## Introduction

Medulloblastoma, the most common solid pediatric tumor, is a devastating central nervous system cancer of the cerebellum that is diagnosed in over 300 children in the US each year^1^. While a standard protocol of surgical resection, chemotherapy, and radiation has improved the five-year survival rate for medulloblastoma patients overall, survivors are left with life-long effects including cognitive deficits and an increased risk of secondary tumors^2^. Many of these sequelae are a direct result of the current aggressive treatment strategy that does not yet account for tumor heterogeneity, highlighting the need to develop targeted, personalized treatments that are both highly effective and less toxic than current therapies.

As in most cancers, there is considerable heterogeneity in medulloblastoma. Classification of tumors based on genetic and phenotypic characteristics has resulted in the division of medulloblastoma into four main subgroups, one of which is driven by aberrant Sonic Hedgehog (SHH) pathway activity^3–5^. The SHH subgroup represents approximately 30% of all medulloblastomas. The overall five-year survival rate for SHH medulloblastoma is fairly high at ~70%. However, patients lacking functional p53 or with metastasis have a worse prognosis, and recurrence of medulloblastoma is uniformly fatal^6–9^. Recently, the SHH subgroup was further sub-divided into four distinct subtypes based on clinical features, DNA methylation status, and gene expression^10^.

The Sonic Hedgehog pathway is critical for embryological development with important roles in patterning, cell differentiation, and organogenesis. Any defects in the SHH pathway can result in severe malformations^11,12^. In cerebellar development, the SHH pathway is essential. SHH is secreted by Purkinje cells in the molecular layer, forming a gradient of SHH ligand that drives the proliferative program responsible for expanding the population of cerebellar granule neuron precursors (CGNPs) in the external granule layer^13^. This program is activated postnatally in mice, and without SHH pathway activity, the population of CGNPs is severely reduced, resulting in a much smaller cerebellum that also lacks proper foliation^13,14^. Importantly, CGNPs are also believed to be the cells of origin for SHH medulloblastoma^15^.

Mechanisms driving cell proliferation are often conserved between development and tumorigenesis, so it is not surprising that in SHH medulloblastoma, the same mitogenic pathway responsible for development of the cerebellum is aberrantly activated through mutations of regulators (*PTCH1*, *SMO*, *SUFU*) or amplification of downstream effectors (*MYCN*, *YAP1*, *GLI*)^3,5^. Aberrant activation of the SHH pathway, combined with other signaling pathways, results in transformation and proliferation of progenitor cells which ultimately form a tumor^13,16–18^. Many cancer types other than medulloblastoma have reported SHH pathway activation including cancers of the lung, pancreas, breast, and prostate in addition to basal cell carcinoma, leukemias, and gliomas^19^. Involvement of the Sonic Hedgehog pathway in so many neoplasms demonstrates that a better understanding of the pathway and the downstream effectors are an important avenue of cancer research^12,20^.

Identification of downstream components of the SHH pathway is an essential part of dissecting Sonic Hedgehog driven carcinogenesis and tumor maintenance and may lead to the discovery of actionable targets for the development of therapeutics. One of the downstream effectors of SHH that is conserved between cerebellar development and medulloblastoma is Yes-associated protein 1 (YAP1)^21^. YAP1 is a transcriptional co-activator that in concert with transcription factors such as TEA domain transcription factor (TEAD) family members, regulates expression of genes in response to signals from upstream pathways including the HIPPO pathway and the SHH pathway^21–26^. Fernandez *et al*. previously established that YAP1 is amplified and highly expressed in SHH medulloblastoma and SHH-N stimulated CGNPs^21^. In that study, YAP1 upregulation was shown to mediate proliferation of CGNPs even in the absence of SHH^21^. In an extension of those findings, using an SHH medulloblastoma mouse model, they demonstrated that tumors with elevated YAP1 expression grew faster and were radioresistant through the upregulation and activation of downstream components that resulted in a bypass of cell cycle checkpoints^25^. Additionally, Dey *et al*. identified a downstream component of YAP1 called Y-box protein 1 (YB1) that is upregulated in SHH stimulated CGNPs as well as in SHH medulloblastoma and is required for cell proliferation in both of these systems^27^.

While pediatric cancer research has made great strides, there is a basic difference between pediatric cancers and adult cancers that has yet to be fully explained. When adult cancers are analyzed, they tend to possess hundreds or even thousands of mutations. In contrast, pediatric cancers including medulloblastoma consistently display a low mutational load^28–30^. In the quest to understand how pediatric cancers form with very few genetic changes, researchers have turned to epigenetics, heritable changes in gene expression without changes to the actual DNA sequence^30,31^. Through methylation of DNA, modifications of histone tails, and chromatin remodeling, epigenetics determines the accessibility of DNA for transcription.

Utilizing the St. Jude Children’s Research Hospital PeCan Data Portal to review a dataset from Paul Northcott comprising 181 SHH medulloblastomas, 65% of the somatic mutations identified were in epigenetic regulators^32^. Importantly, epigenetic regulation is reversible and could be a targetable approach to improve medulloblastoma treatment outcomes^30^. With this in mind, we sought to know whether SHH altered the expression levels of any epigenetic regulators. As detailed in our results, we identified the chromatin remodeler called helicase, lymphoid-specific (HELLS) as a gene with increased expression in SHH stimulated CGNPs.

HELLS protein, also referred to as LSH, PASG, or SMARCA6 is a member of the SNF2 family of chromatin remodelers, ATP-dependent enzymes that modify nucleosome organization and position by disrupting histone-DNA interactions, thereby regulating access to the DNA^33^. While most members of this family accomplish this as a part of a large complex, HELLS has not been identified as part of such a complex^34^. However, binding of HELLS to DNMT3B and co-immunoprecipitation of HELLS with DNMT1, HDAC1, and HDAC2 has been reported^35^. Roles for HELLS in development, epigenetics, senescence, DNA repair, transcription, stem cell maintenance, *HOX* gene control, and meiosis have been proposed^36–42^. Importantly, homozygous deletion of *Hells* is perinatally lethal in mice and a hypomorphic mutation of *Hells* results in the short-term survival of a few pups which exhibit growth retardation, a premature aging phenotype, and early mortality^37,43–46^. The *HELLS* gene is highly conserved, with murine and human HELLS sharing 95% protein homology^45^. Aside from the thymus and testis, expression of *HELLS* in normal adult cells is low and the higher expression of *HELLS* observed in stem cells decreases upon differentiation^45,47,48^. While there are many reports of HELLS functions in various conditions, there is very little known about how HELLS is regulated. Transcriptional activation of HELLS by E2F1 in retinoblastoma, gliomas, and in cell lines has been reported while in skin stem cells HELLS has been reported to be a target of an isoform of p63, but there are no previous reports of HELLS regulation in medulloblastoma^39,49–51^.

With so many possible functions of HELLS that result in alterations in gene expression, knowing it is expressed at higher levels in CGNPs stimulated with SHH-N led us to hypothesize HELLS involvement in cerebellar development and medulloblastoma. Here, we investigated HELLS expression and regulation in murine cerebellar development and SHH medulloblastoma. We found significantly higher expression of *HELLS* in the developing murine cerebellum compared to the mature cerebellum and the cerebral cortex. Additionally, we observed higher levels of HELLS in mouse SHH medulloblastoma tumors when compared to adjacent non-tumor cerebellum. We also show evidence that this upregulation is driven by the SHH pathway through the downstream effector YAP1. These novel findings add to our understanding of the downstream activity of the SHH pathway and may help bridge the gap between the downstream effector YAP1 and the unregulated proliferation in SHH medulloblastoma.

## Results

### HELLS expression is upregulated in SHH-N stimulated CGNPs and in the developing murine cerebellum

**Figure 1:**
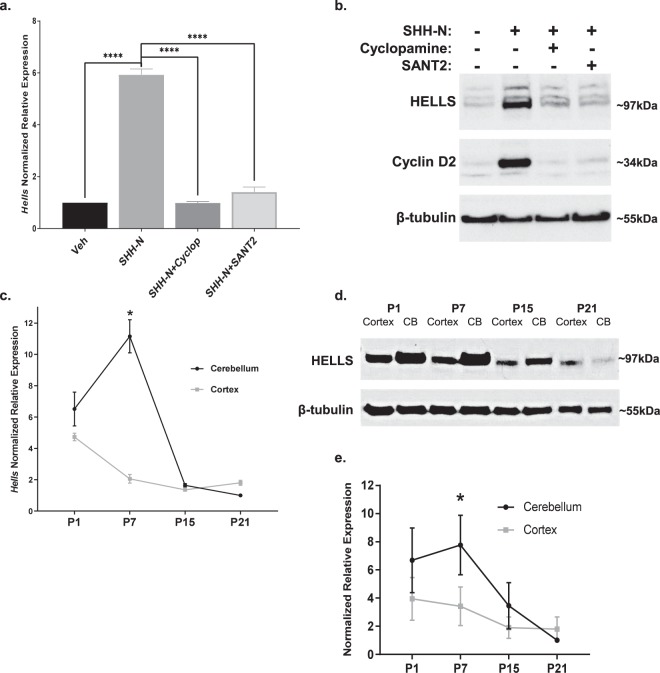
HELLS is upregulated in SHH-N stimulated CGNPs and the developing mouse cerebellum. (**a**) qRT-PCR analysis of SHH-N stimulated CGNPs show increased *Hells* expression at 48 hours which is abrogated by inhibition of SMO with either cyclopamine or SANT2. N = 3, one-way ANOVA with Dunnett’s Multiple Comparison, ****P < 0.0001; Data represent mean ± S.E.M. (**b**) Western blot analysis of protein levels of HELLS for the same experiments depicted in 1a. Cyclin D2 is shown as an indicator of SHH signaling^87^. Data are representative of 3 independent replicates. Full-length blots are presented in Supplementary Fig. S4. (**c**) qRT-PCR analysis illustrates *Hells* mRNA levels in the cerebella of P7 mice compared to the cerebral cortex or the cerebella at other time points. Normalization to P21 cerebellum. N = 3, two-tailed paired t-test, *P < 0.05; Data represent mean ± S.E.M. (**d**) Western blot for protein levels of HELLS in the murine cerebella of during postnatal development. Data are representative of three separate experiments. (**e**) Densitometric analysis of western blots depicted in panel d. Protein bands were quantitated by densitometry, relative to β-tubulin, and normalized to P21 cerebellum. N = 3, two-tailed paired t-test, ***P < 0.001. Data represent mean ± S.E.M. Western blots are cropped to show bands of interest clearly. Bands presented together in an image are from the same gel/blot. Full-length blots are presented in Supplementary Fig. S4.

Cerebellar granule neural precursors, the putative cell of origin for SHH medulloblastoma, can be isolated from the cerebella of 4–5-day old mice and grown in serum-free culture for 3–4 days before they begin to differentiate, even with SHH in the culture medium. These cells provide an important model as the SHH induced mitogenic signaling and gene expression seen in these cells is similar to that seen during cerebellar development and in SHH medulloblastoma^52^. Microarray experiments were done on CGNPs in the presence or absence of SHH-N, the recombinantly produced, biologically active N-terminal fragment of SHH, which revealed genes regulated by SHH in our system. This data included several epigenetic factors that seem to be upregulated in CGNPs cultured with SHH-N. One of these factors is the chromatin remodeler HELLS. HELLS was also identified as one of the genes upregulated by SHH-N in a 2003 study from the Wechsler-Reya lab^53^. Validation with qRT-PCR confirmed that *Hells* mRNA expression is consistently upregulated in SHH treated CGNPs. A statistically significant 5.9 ± 0.2 fold increase (mean ± S.E.M, N = 3) in *Hells* mRNA is observed in CGNPs with SHH-N stimulation while expression returns to basal levels with the addition of either cyclopamine or SANT-2^54^, which both directly inhibit Smoothened (SMO) with different mechanisms of action (Fig. 1a). This differential regulation is also demonstrated in HELLS protein levels (Fig. 1b).

In mice, the cerebellum at birth is a small cluster of cells, but within 15 days, massive proliferation has taken place as well as foliation, resulting in a fully formed cerebellum^14^. Expansion of this cell population peaks between postnatal days five and eight (P5-P8) and is driven by SHH signaling^55–59^. Since HELLS regulation seems to require SHH signaling in our systems and this pathway is critical to postnatal cerebellar development, we speculated that HELLS could be a part of the SHH proliferative program. To evaluate endogenous levels of HELLS in the developing murine brain, pooled tissue samples from three separate litters were collected at multiple time points during normal murine development and then analyzed for mRNA expression and protein level of HELLS. We establish here that HELLS is significantly higher in the P7 murine cerebellum when compared to the mature cerebellum and the P7 cerebral cortex. As illustrated in Fig. 1c, cerebellar *Hells* mRNA levels are highest at P7, while in the cerebral cortex *Hells* mRNA levels are highest at P1, but still lower than in the cerebellum. In keeping with the results seen in expression, HELLS protein levels (Fig. 1d) in the cerebellum are highest at P7 (n = 3). Densitometry provided in Fig. 1e indicates HELLS protein level in the P7 cerebellum is 7.8 ± 0.8 fold higher than in P21 cerebellum (mean ± S.E.M., N = 3). HELLS protein level is 2.5 ± 0.1 times higher in the P7 cerebellum compared to the P7 cortex (mean ± S.E.M., N = 3). The timing of the highest levels of HELLS coincides with highly active SHH signaling and CGNP proliferation, suggesting that HELLS may have a role in SHH dependent postnatal cerebellar development^55,56^.

### Elevated expression of HELLS in Human SHH medulloblastoma

**Figure 2:**
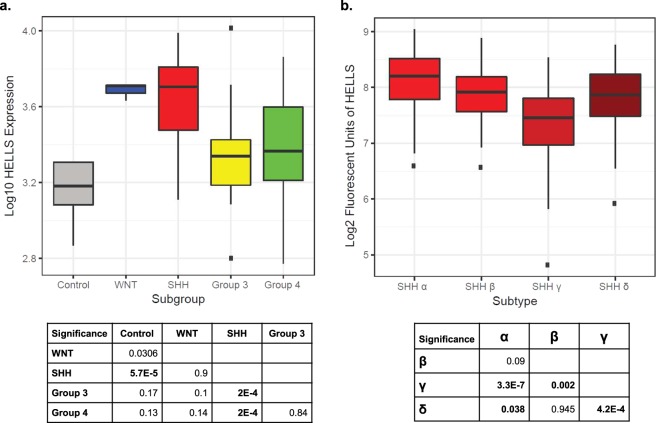
*HELLS* is upregulated in human SHH medulloblastoma. (**a**) Microarray data showing *HELLS* expression levels across medulloblastoma subgroups. Subgrouping of these samples was done using nanostring. Statistical significance comparisons are displayed on the table below the figure and were performed using pairwise t-tests and corrected using the Benjamini-Hochberg adjustment. This figure represents data published in Vladoui 2018^88^. (**b**) Further analysis of the Cavalli *et al*. 2017 dataset allowed visualization of *HELLS* expression in the SHH subtypes. Differential expression was determined using pairwise t-tests and corrected using Benjamini-Hochberg adjustment on the nonlog-transformed values. Subtypes were determined using SNF clustering of gene expression and methylation profiles.

Developmental pathways such as SHH are often co-opted by cancer, and this is true for SHH medulloblastoma. Since we observed increased levels of HELLS in SHH stimulated CGNPs, and in the developing murine cerebella, we next sought to determine whether *HELLS* is upregulated in human medulloblastoma. To this end, we queried a database of human medulloblastoma samples originally made public as part of the Stand Up 2 Cancer project under the accession number EGAS00001000273. Along with having samples across all four subgroups, the dataset also includes control samples including five fetal and four adult samples. As shown in Fig. 2a, compared to normal cerebellum, *HELLS* is expressed at higher levels in all medulloblastomas with the highest expression in the SHH subgroup. Significant differences between the control samples and the WNT and SHH subgroups are shown in the table accompanying Fig. 2a. Significant differences are also apparent between the SHH subgroup and subgroups 3 and 4.

In 2017, the four subgroups of medulloblastoma were further categorized into subtypes^10^. In the SHH group, four subtypes were identified based on similarity network fusion of genome-wide DNA methylation and gene expression data which was correlated with clinical and genomic characteristics. The SHH α subtype is comprised of children aged 3–16 years with a higher percentage of *MYCN*, *GLI2*, and *YAP1* amplifications. In SHH α, mutations in *TP53* were prognostic, conferring a survival disadvantage. Consequently, SHH α is the subtype with the worst prognosis^10^. Infant SHH medulloblastoma patients largely fall within the SHH β and SHH γ subtypes with the β subtype exhibiting higher rates of metastatic disease, deletions of tumor suppressor *PTEN*, and worse outcomes than SHH γ. Lastly, the SHH δ subtype is primarily adults with better overall outcomes and is the subtype with the highest percentage of TERT promoter mutations^10^. Further analysis of the Cavalli 2017 dataset allowed us to visualize HELLS expression in the SHH subtypes (Fig. 2b). Interestingly, HELLS expression is highest in the SHH α subtype and lowest in subtype γ. While these differences are statistically significant as shown in the table provided, HELLS is elevated in all SHH medulloblastomas.

### HELLS levels are increased in SHH murine medulloblastoma

**Figure 3:**
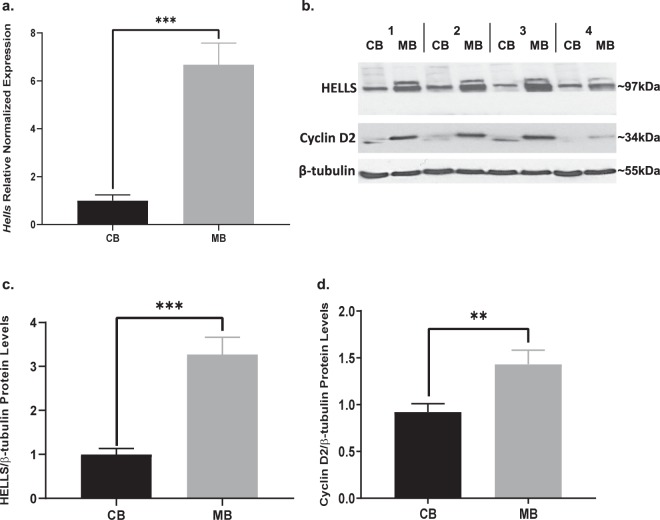
HELLS is upregulated in SHH murine medulloblastoma. (**a**) *Hells* mRNA transcript levels in *NeuroD2:SmoA1* mouse medulloblastoma (MB) and adjacent non-tumor cerebellum (CB). Expression levels were analyzed normalized by average expression of adjacent non-tumor cerebellum. N = 9; two-tailed paired t-test, ***P < 0.001. Data represent mean ± S.E.M. (**b**) Western blot depicting protein levels of HELLS in murine *NeuroD2:SmoA1* MB and adjacent non-tumor CB. Blot shows HELLS levels in four separate MB – CB pairs. A blot showing four additional tumors is provided in Supplementary Fig S1. (**c**) Densitometric analysis of HELLS western blot depicted in panel b. Protein bands were quantitated by densitometry, relative to β-tubulin, and normalized to adjacent non-tumor cerebellum. N = 8, two-tailed paired t-test, ***P < 0.001. Data represent mean ± S.E.M. (**d**) Densitometric analysis of Cyclin D2 western blot depicted in panel b. Protein bands were quantitated by densitometry, relative to β-tubulin, and normalized to adjacent non-tumor cerebellum. N = 8, two-tailed paired t-test, **P < 0.01. Data represent mean ± S.E.M. Western blots are cropped to show bands of interest clearly. Bands presented together in an image are from the same gel/blot. Full-length blots are presented in Supplementary Fig. S1.

SHH medulloblastoma is characterized by aberrations in the SHH pathway, so our next question was to determine whether HELLS is upregulated in an SHH medulloblastoma mouse model. We use the *NeuroD2:SmoA1* model originally developed by Jim Olson’s research group^60^. In these mice, a point mutation of the Smoothened (*Smo*) gene driven by the Neuro D2 promoter results in a SMO protein that is constitutively activated, generating downstream sustained high SHH signaling. Approximately 70% of these mice develop tumors between four and six months of age. Upon symptom onsets such as ataxia or other neurological signs, the tumor and adjacent non-tumor cerebellar tissue can be collected for analyses. As shown in Fig. 3a, a comparison of mouse medulloblastoma tumors and adjacent non-tumor cerebellum reveals an average 6.7 ± 0.9 (mean ± S.E.M., N = 9) increase in the expression of *Hells* mRNA in these tumors. This increase can also be seen in the level of HELLS protein from eight separate SHH murine tumors compared to normal adjacent cerebellum (Fig. 3b and Supplementary Fig. S1). Densitometry and statistical analysis indicate 3.3 ± 0.4 (mean ± S.E.M., N = 8) higher levels of HELLS protein in *NeuroD2:SmoA1* mouse medulloblastoma tumors (Fig. 3c). Protein levels of Cyclin D2 are also elevated, as would be expected in tumor tissue (Fig. 3d).

### HELLS expression is dependent on YAP1 activity

In our experimental systems, both mRNA expression and protein levels of HELLS are modulated by the SHH pathway. Downstream of SMO there is more than one possible effector of the pathway, so we next sought to determine which of these effectors might be implicated in HELLS regulation. The canonical SHH pathway transcriptional program is carried out by GLI proteins. Therefore we started by investigating HELLS regulation by GLI. The GLI family of proteins are transcription factors with targets including *PTCH1*, *PTCH2*, and *GLI1*^16^. GLI1 and GLI2 have both been implicated in SHH medulloblastoma and efforts to inhibit them are ongoing^19^. The small molecule GANT61 inhibits both GLI1 and GLI2 at an IC50 of ~5 µM in the NIH 3T3 cell line^61^. Using GANT61 at a range of doses in CGNPs and MBCs we observed slight downregulation of HELLS, along with concurrent increases in cleaved caspase 3 (Supplementary Fig. S2). This led us to conclude that this downregulation is due to cell death processes as opposed to direct regulation of HELLS by GLI proteins.

**Figure 4:**
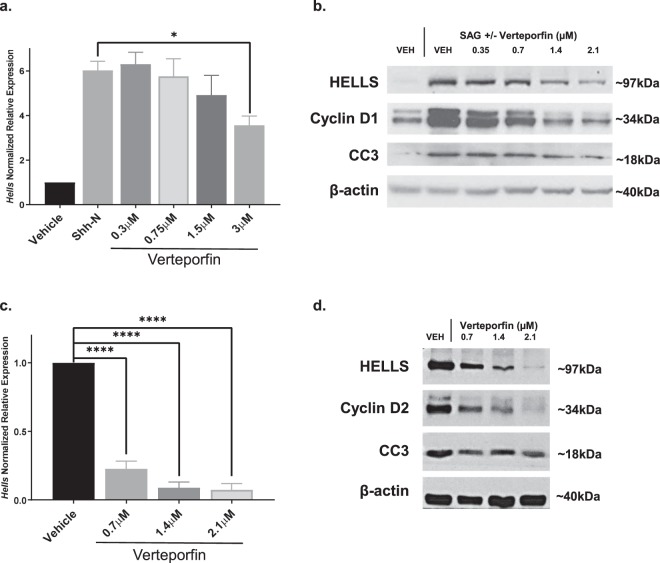
HELLS expression and protein levels are modulated with YAP1/TEAD inhibition downstream of SHH signaling. (**a**) *Hells* mRNA expression in SHH-N or SAG stimulated CGNPs treated with increasing concentrations of the YAP1/TEAD inhibitor verteporfin. N = 3, one-way ANOVA with Dunnett’s Multiple Comparison, *P < 0.05. Data represent mean ± S.E.M. (**b**) Western blot showing protein levels of HELLS, cleaved caspase 3 (CC3), and Cyclin D1 in verteporfin treated CGNPs. Blot is representative of three replicates. Full-length blots are presented in Supplementary Fig. S4. (**c**) *Hells* mRNA expression in cultured murine medulloblastoma cells (MBCs) treated with increasing doses of verteporfin. N = 3, one-way ANOVA with Dunnett’s Multiple Comparison, ****P < 0.0001. Data represent mean ± S.E.M. (**d**) Western blot showing protein levels of HELLS, cleaved caspase 3 (CC3), and Cyclin D2 in verteporfin treated MBCs. Western blots are cropped to show bands of interest clearly. Bands presented together in an image are from the same gel/blot. Full-length blots are presented in Supplementary Figs S4 and S5.

We next asked whether HELLS may lie downstream of YAP1, a transcriptional coactivator known to be upregulated in SHH medulloblastoma^21^. TEAD proteins are among the transcriptional partners of YAP1, and the interaction between TEAD and YAP1 can be inhibited with verteporfin, a drug used clinically with light activation to treat macular degeneration^62^. In multiple studies, verteporfin has also been shown to inhibit the YAP1/TEAD interaction without light activation^62–66^. *Hells* expression in SHH stimulated CGNPs was inhibited in a dose-dependent manner when cells were treated with verteporfin (Fig. 4a). This inhibition was also seen at the protein level with no increase in cleaved caspase 3 levels (Fig. 4b). This suggests verteporfin specifically downregulates HELLS via disruption of the YAP1/TEAD complex as opposed to causing the cells to undergo programmed cell death. We also observed a reduction in Cyclin D1, indicating a reduction in the proliferative potential of these cells. To assess verteporfin effects in the context of tumor cells, we cultured medulloblastoma cells (MBCs) dissociated from *NeuroD2:SmoA1* murine tumors. In MBCs, the downregulation of HELLS expression with verteporfin is even more pronounced (Fig. 4c,d). This downregulation was seen at both the mRNA and protein levels and importantly, the levels of cleaved caspase 3 were not affected. Additionally, there is a concurrent reduction of Cyclin D2 with the reduction of HELLS in response to verteporfin inhibition of the YAP1/TEAD interaction (Fig. 4d).

**Figure 5:**
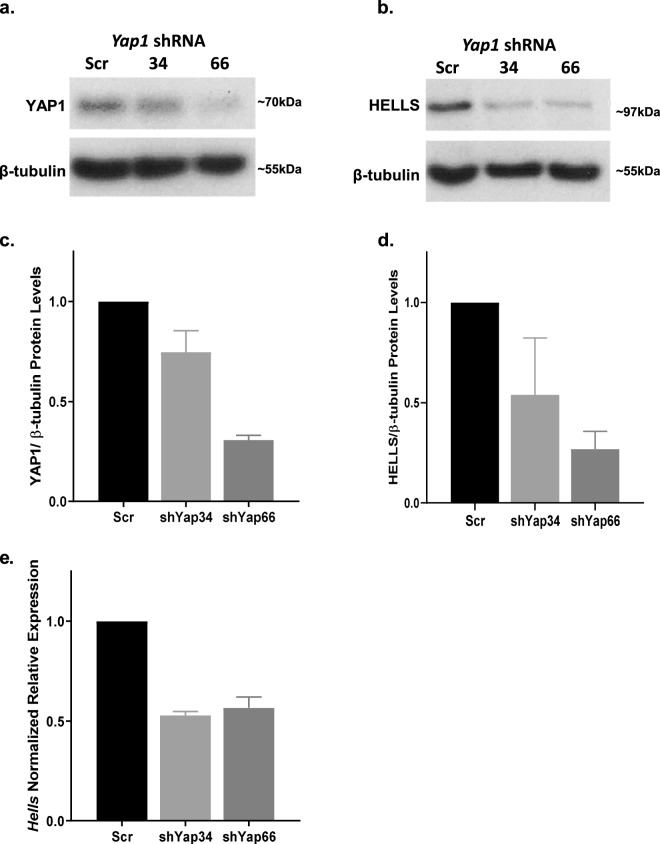
Knockdown of YAP1 results in a reduction of HELLS mRNA and protein levels. (**a**) Western blot illustrating YAP1 protein levels in murine medulloblastoma slice culture electroporated with scrambled shRNA (Scr) or two separate *Yap1* shRNAs (34 or 66). (**b**) Western blot of HELLS protein levels in the same experiment depicted in (**a**). (**c**,**d**) Densitometric analysis of western blots depicted in panels a and b. Protein bands were quantitated by densitometry, relative to β-tubulin, and normalized to Scr. N = 2. Data represent mean ± S.E.M. (**e**) qRT-PCR analysis of *Hells* mRNA expression with knockdown of *Yap1*.

In addition to pharmacological inhibition of the interaction between YAP1/TEAD, we sought to determine if depletion of YAP1 would alter the levels of HELLS in our systems. Utilizing the *NeuroD2:SmoA1* mouse model, we performed electroporation of tumor slices with plasmid DNA for either a scrambled shRNA or two separate YAP1 shRNAs. We found that protein levels of YAP1 were reduced with YAP1 shRNAs, resulting in a reduction of HELLS protein levels (Fig. 5a–d). Additionally, qRT-PCR demonstrated lower levels of *Hells* transcripts with shRNA knockdown of YAP1 (Fig. 5e). Together, these experiments suggest regulation of HELLS through YAP1, but cannot tell us whether *Hells* is a direct target of the YAP1/TEAD transcriptional program.

### YAP1/TEAD binding to DNA upstream of HELLS

**Figure 6:**
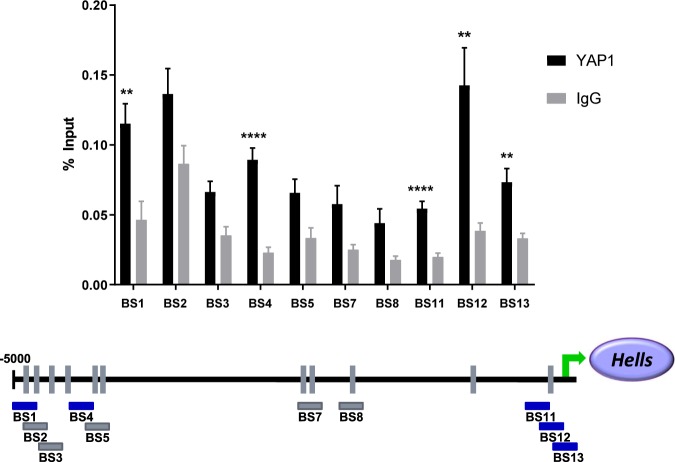
Binding of YAP1/TEAD to *Hells* upstream DNA. Putative TEAD binding sites up to 5000 bp upstream of *Hells* transcription start site were identified. Tiled primers to those locations were designed and used to interrogate DNA isolated during chromatin immunoprecipitation using a YAP1 antibody. IgG was used as control. Values are presented as a percentage of input (non-immunoprecipitated chromatin). Data represent mean ± S.E.M. t-tests were used to compare YAP1 to IgG at each putative binding site, and the two-stage linear step-up procedure of Benjamini, Krieger, and Yekutieli was applied to avoid false discovery. N = 8 (BS1 *P* = 0.005; BS2 *P* = 0.053; BS3 *P* = 0.013; BS4 *P* = 0.00003; BS5 *P* = 0.025; BS7 *P* = 0.055; BS8 *P* = 0.041; BS11 *P* = 0.00009; BS12 *P* = 0.004; BS13 *P* = 0.004).

To further evaluate possible YAP1 regulation of HELLS downstream of SHH, we carried out chromatin immunoprecipitation (ChIP) followed by quantitative PCR. For these experiments, we utilized the PZp53 murine medulloblastoma cell line, a line derived from a mouse medulloblastoma heterozygous for PTCH1 and null for p53^67^. First, we verified that these cells expressed HELLS and that HELLS was regulated in a manner similar to what we observed in CGNPs and MBCs (Supplementary Fig. S3). Protein and DNA from crosslinked cells were immunoprecipitated using a YAP1 antibody, and the resulting DNA was queried with primers designed to recognize putative upstream TEAD binding sites as YAP1 itself does not bind directly to DNA. Using the Eukaryotic Promoter Database, putative TEAD binding sites were identified up to 5000 base pairs upstream of the HELLS transcription start site (TSS). As illustrated in Fig. 6, five different binding sites were significantly enriched for YAP1/TEAD binding. Two of the binding sites are distal to the TSS and may represent binding to an enhancer as the transcriptional activity of YAP1 has been identified as being mediated by binding of TEAD to distal enhancers^23^. The other three sites enriched for YAP1/TEAD are located in a region identified as the HELLS promoter region. These experiments indicate YAP1/TEAD binding to HELLS upstream DNA, suggesting direct regulation of HELLS transcription by YAP1/TEAD in SHH medulloblastoma.

## Discussion

**Figure 7:**
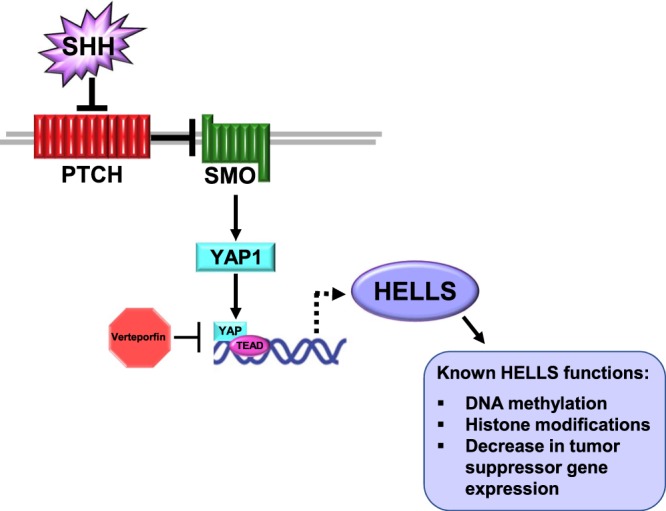
Model of SHH induced increase of HELLS. Activation of the SHH pathway leads to upregulation and activation of YAP1, a transcriptional co-activator that functions in partnership with TEAD family transcription factors. We propose that *HELLS* is a YAP1/TEAD transcriptional target. *HELLS* is upregulated in SHH-N stimulated CGNPs and in murine and human SHH medulloblastoma. This upregulation can be modulated by inhibition of the interaction between YAP1 and TEAD family transcription factors using the drug verteporfin or by knockdown of YAP1. Chromatin immunoprecipitation indicates YAP1/TEAD binds to DNA upstream of *Hells* suggesting that regulation of *Hells* transcription through YAP1 may be direct.

Here we have identified YAP1 mediated, Sonic hedgehog induced upregulation of HELLS in murine models of cerebellar development and SHH medulloblastoma. In SHH stimulated precursor cells and SHH medulloblastoma cell culture, HELLS is upregulated at both the mRNA and protein levels. Analysis of the developing murine brain reveals elevated HELLS expression in the cerebellum compared to the neural cortex in keeping with the timing of high levels of SHH activity and CGNP proliferation. We observe upregulated HELLS expression in both murine and human SHH medulloblastoma. In murine primary cell culture, this upregulation can be abolished by pharmacological inhibition of the SHH effector YAP1, suggesting regulation through this downstream effector. The effects on HELLS protein level appear to be greater than those on the mRNA level. While we frequently do not observe a direct correlation between levels of mRNA and protein, it is also possible that other aspects of YAP1 activity or YAP1 downstream effectors are affected by inhibition with verteporfin and that these effects regulate HELLS translation and/or stabilization. However, knockdown of YAP1 in murine medulloblastoma slice culture also resulted in decreased levels of HELLS mRNA and protein, indicating that HELLS is regulated through YAP1. Furthermore, chromatin immunoprecipitation suggests direct regulation of HELLS through YAP1. Our results indicate a possible role for HELLS in the proliferative program of SHH in both development and medulloblastoma (Fig. 7). Indeed, the verteporfin induced decrease in CyclinD1 or D2 in CGNPs and MBCs links the YAP1/HELLS axis to a requirement for proliferation in the developing cerebellum and SHH medulloblastoma tumor cells.

Epigenetics is becoming an increasingly important area of cancer research, particularly in pediatric malignancies where the low mutational burden leaves researchers looking for viable targets. Inhibitors of drugs targeting epigenetic factors have been approved for use in several cancers, and clinical trials are ongoing in several other types of cancer^68^. A more complete understanding of the epigenetic underpinnings of medulloblastoma could provide valuable avenues of research for therapeutic interventions that have the potential to be more effective and less harmful than our current non-specific DNA damaging radiation and chemotherapeutics. As a member of a family of chromatin remodelers with putative roles in senescence^41,44,69,70^, repression of tumor suppressor genes^41,44^, stem cell control^38,45,48,51^, Hox gene control^71^, and DNA repair^72^, our identification of HELLS upregulation in medulloblastoma could be an important finding. That this upregulation is a result of SHH pathway activation and HELLS is also upregulated in normal murine cerebellar development adds weight to this theory.

HELLS is known to be an important epigenetic regulator, linking multiple mechanisms for chromatin remodeling including methylation of DNA and modification of histone proteins^38^. In embryological development, HELLS is vital for normal murine development with HELLS null pups dying at or shortly after birth with severe renal lesions^73^. A mouse model with a hypomorphic mutation resulted in some mice surviving, but with growth retardation, premature aging, and early death^44^. Coupled with these phenotypic results in mice with a truncated HELLS protein, upregulation of tumor suppressor genes including p16, p21, and p53 was observed, leading to replicative senescence^44^. Less is known regarding HELLS effects in humans, but it is known that mutations of HELLS cause ICF syndrome (immunodeficiency, centromeric instability, facial anomalies) and that HELLS forms a nucleosome remodeling complex with the protein Cell Division Cycle Associated 7 that is often defective in this disease^74^. Utilizing the R2 web-based genomics analysis and visualization application to analyze human gene expression in a large cohort of human medulloblastomas from Cavalli 2017^10^ allowed us to evaluate HELLS gene expression correlation in SHH medulloblastoma. This analysis revealed highly significant correlations for genes involved in the cell cycle and DNA repair (Table 1). Developmentally, as cerebellar formation occurs predominantly postnatally in mice the effects of HELLS depletion on that process would be difficult to assess as Hells-/- pups die at birth, but a 2017 report from Yan *et al*. using neural stem/progenitor cells showed the importance of HELLS in mouse neural development with reduced growth of these cells, increased apoptosis, and impaired ability of self-renewal^45^.

**Table 1:** *HELLS* gene expression correlation in SHH medulloblastoma.

Mini Ontology Analysis				
Group	InSet	Total	%	pval
All	3378	8836	38.20%	1
**DNA repair**	122	192	63.50%	**5**.**30E-13**
TF	284	783	36.30%	0.26
Apoptosis	239	595	40.20%	0.33
**cell cycle**	281	467	60.20%	**1**.**70E-22**
Development	552	1519	36.30%	0.13
Diff	226	639	35.40%	0.14
Drugtarget	423	1098	38.50%	0.84
**Kinase**	274	623	44.00%	**3**.**10E-03**
**Membrane**	1692	4691	36.10%	**2**.**30E-03**
**sign transd**	1033	2932	35.20%	**8**.**40E-04**
transcription regulator Act	432	1162	37.20%	0.46
transcriptional repressor Act	82	175	46.90%	0.02

Using R2, a web-based application for genomics analysis, the correlation between *HELLS* expression and the expression of other genes was evaluated in a human dataset from Cavalli *et al*.^10^. Focusing on the 223 SHH medulloblastoma samples in their dataset, correlations with various gene groups are listed with the corresponding significance of the relationship. Sourcegene = HELLS(7929438); *p*-value correction for multiple testing: False Discovery Rate; ‘R2: Genomics Analysis and Visualization Platform (http://r2.amc.nl)’.

In SHH-N stimulated CGNPs and in normal murine cerebella with endogenous SHH expression, HELLS upregulation is observed in these highly proliferative cells. As HELLS is known to suppress inhibitors of the cell cycle^44^, it is possible that increased HELLS levels in these cells enables continued cell cycle activity and proliferation. Future work to dissect HELLS activity in these conditions may provide a better understanding of HELLS role in cerebellar development.

Involvement of HELLS has been reported in a number of different cancers including leukemia^75^, retinoblastoma^39^, lung cancer^76^, gliomas^49^, head and neck cancer^77,78^, breast cancer^79^, prostate cancer^80^, and skin cancers^51^. In some of these cancers, mutations are present, resulting in increased HELLS levels or activity, however, in other cases HELLS levels are decreased or its function is abrogated. For example, Benavente *et al*. found that HELLS was tumorigenic only when upregulated in the tumor cell of origin and that HELLS upregulation epigenetically mediated the aberrant expression of genes that drive retinoblastoma tumorigenesis while a recent finding in osteosarcoma found that HELLS is non-essential for tumorigenesis in this cancer^39,81^. These examples illustrate the importance of context when attempting to unravel the implications of gene expression in cancer.

HELLS regulation of epigenetic factors is itself normally tightly regulated, and alterations in either direction result in deleterious effects^73,80,82^. Expression of HELLS in non-tumor cerebellar cells is low or non-existent, making HELLS an attractive, tumor-specific target for research. That HELLS expression in MBCs was abrogated to a greater extent than in CGNPs with lower doses of verteporfin seems to confirm the tumor-specific importance of HELLS. As for the role of HELLS in SHH medulloblastoma, important work done in the Charron lab found that evasion of senescence was a crucial step in SHH medulloblastoma formation^83^. This escape was facilitated by the loss of p53 or inactivation of p16ink4a and they identified higher p16 regulatory sequence methylation in 22% of the human SHH medulloblastomas they analyzed^83^. Previous work from other groups showed cell senescence and upregulation of tumor suppressor genes including p16, p21, and p53 in a HELLS mutant mouse model^44^. Future studies of the specific effects of HELLS in SHH medulloblastoma could provide a connection between the elevated HELLS levels we have identified, the known effects of HELLS on tumor suppressors, and the p16 inactivation induced evasion of senescence demonstrated in SHH medulloblastoma.

YAP1 overexpression in SHH medulloblastoma allows cancer cells to bypass cell cycle checkpoints by upregulating and activating downstream components^25^. While some of those components have been discovered, there are others yet to be identified. Our finding of YAP1 directed HELLS upregulation in SHH medulloblastoma suggests a role for HELLS regulated chromatin remodeling in SHH medulloblastoma. As other epigenetic regulators have been targeted for cancer treatment with varying degrees of success, future studies to determine HELLS role in SHH medulloblastoma may lead to new avenues of research and eventually to new therapeutic interventions.

## Methods

### Animal studies

All animal studies were carried out in accordance with the Emory University Institutional Animal Care and Use Committee guidelines under Dr. Kenney’s approved IACUC protocol #2003395 “Interactions between signaling pathways in the developing brain and medulloblastoma”. *NeuroD2:SmoA1* mice were purchased from Jackson Labs (008831); CD-1 mice were purchased from Charles River; *NeuroD2:SmoA1* and *Atoh1:GFP* mice were crossed to generate *NeuroD2:SmoA1;Atoh1:GFP*, in which all tumor cells produce GFP protein^84^. For studies involving HELLS in murine brain development, pooled tissue samples from three separate litters of CD-1 mice were used.

### Patient data analysis

#### RNA Seq samples

Patient data analyzed for HELLS RNA expression is available. A total of 105 patients samples were subgrouped into 3 WNT-activated, 49 SHH-activated, 19 Group 3, 25 Group 4 medulloblastomas, as well as control samples including five adult cerebella samples and four fetal cerebella samples. RNA sequencing data is available via the European Genome-phenome Archive (EGA; https://www.ebi.ac.uk/ega/home) accession number EGAD00001004435. Fastq files were processed using the STAR alignment tool^85^ and normalized using the DESeq. 2 package^86^ based upon the hg19 reference genome and the GENCODE v19 gene annotation set. Differential expression of the HELLS gene was determined using pairwise t-tests and corrected using Benjamini-Hochberg adjustment. Gene transcript-level expression was converted to log10 normalized values for illustrating box plots.

#### Microarray samples

A total of 187 SHH samples that were subtyped into 53 alpha, 31 beta, 37 gamma, 66 delta were used and analyzed for *HELLS* microarray expression under the accession number GSE85217. Raw microarray sequencing data is also available for this dataset via the Gene Expression Omnibus (GEO) accession number GSE85218; however, the publicly available normalized matrix was used.

### Cell culture

CGNPs were isolated and cultured as previously published^87^. Briefly, cerebella from 5-day old CD-1 mice were harvested, cells were disassociated into a single cell suspension, plates were coated with poly-DL-ornithine, and cells were plated in DMEM-F12/N2 with or without recombinant SHH-N at 3 µg mL^−1^ or SAG at 200 nM to activate the SHH pathway.

MBCs were isolated from *NeuroD2:SmoA1* and *NeuroD2:SmoA1;Atoh1:GFP* murine tumor tissue. Tumors were disassociated into single cell suspensions using incubation with Papain/DNase, filtered through a cell strainer, and then separated by centrifugation through 35/65% Percoll (GE Healthcare) and cells at the interphase were collected for plating. MBC plates were coated with poly-D-lysine (Sigma) and Geltrex (Gibco) and cells were grown in Neurobasal medium with B27 supplement (without vitamin A), Glutamax, sodium pyruvate, and 1% penicillin-streptomycin (all from Gibco).

The PZp53 cell line was used for ChIP experiments. This cell line is derived from a *Ptc*^+/−^/*p53*^−/−^ murine medulloblastoma. The SHH pathway is constitutively active in this cell line, and it is useful in evaluating downstream effects on the pathway. PZp53 cells were cultured in DMEM with 10% FBS.

Drug treatment of cells was done 24 hours after cells were plated and cells were allowed to grow another 48 hours in the presence of vehicle or drug. Cyclopamine (R&D Systems) was utilized at 1 µg mL^−1^. SANT2 (Alexis BioChemicals) was used at a concentration of 100 nM. GANT61 (A kind gift from Dolores Hambardzumyan; synthesized at Memorial Sloan Kettering) was resuspended in ethanol to a concentration of 10 mM and stored at −80 °C until ready for use. Verteporfin (Sigma) was dissolved in DMSO to a concentration of 1.4 mM and stored at −20 °C prior to use. GANT61 and verteporfin treatments were used at the indicated doses.

### Tumor slice culture

Tumors were isolated from *NeuroD2:SmoA1* or *NeuroD2:SmoA1;Atoh1:GFP* mice and divided into small (~3 mm) pieces. Tumor pieces were embedded into 4% low melt agarose in sterile 1X HBSS at ~37 °C. Agarose embedded tumor was maintained on ice throughout the remainder of the protocol. Tumor blocks were sliced into 300 µm slices using a Leica V1200S vibratome at amplitude 0.75 mm and speed 0.50 mm s^−1^. Slices were maintained in cold 1X HBSS with penicillin and streptomycin until electroporation was completed. Tumor slices were electroporated with 2.5 µg of DNA (shRNA scrambled (SCR), *Yap1* shRNA 34 (MISSION shRNA TRCN0000238434), or *Yap1* shRNA 66 (MISSION shRNA TRCN0000095866)) using a BTX ECM830 electroporator (Pulse: 5 × 5 ms, Interval: 995 ms, Voltage: 25 V). Slices were then placed in culture in 6 well plates with EMD Millipore PICM03050 inserts in 1 mL DMEM-F12 with N2 supplement and penicillin/streptomycin. Slices were cultured for ~72 hours with media changed every 24 hours. Tumor slice cultures were collected and processed for the evaluation of mRNA and protein levels.

### RNA extraction and qRT-PCR

RNA extraction from cells and tissues was done according to the protocol provided by Genecopeia. Briefly, cells or tissues were homogenized in Trizol (ThermoFisher), phase separation was done using chloroform and centrifugation, and RNA was precipitated with alcohol washes. RNA quantification was done with a Nanodrop 1000. cDNA first strand synthesis was done using the High-Capacity cDNA Reverse Transcription Kit from Applied Biosystems. Primers for *Hells* were purchased from GeneCopoeia, Inc. Rockville, MD (Primer ID: Mm-QRP-21432). Primers for controls *Gli1* (Unique Assay ID: qMmuCID0026119), *Gli2* (Unique Assay ID: qMmuCID0005725), *CyclinD2* (Unique Assay ID: qMmuCID0023538), *B2M* (Unique Assay ID: qMmuCID0040553), and *GusB* (Unique Assay ID:qMmuCID0046361) were purchased from Bio-Rad. Quantitative real-time PCR (qRT-PCR) was done in triplicate in 96 well plates using the SsoAdvanced™ Universal SYBR® Green Supermix (BioRad) and the C1000 Touch™ Thermal Cycler with the CFX96™ Optical Reaction Module from BioRad. At least three biological replicates were done for each experiment, and the results were processed for statistical analysis using the BioRad CFX Manager™ software and Prism (GraphPad).

### Protein preparation and immunoblotting

Proteins were isolated from cells and tissues using the following methods. Cells from cell culture were washed with phosphate-buffered saline and collected in lysis buffer and processed as previously outlined^87^. Protein content was determined using the BioRad protein assay. Equal amounts of whole protein lysate were separated on SDS-polyacrylamide gels and transferred to activated polyvinylidene fluoride membranes (Millipore) Western blotting was done in keeping with standard protocols. Primary antibodies used were: anti-HELLS (ABD41, Millipore), anti-Cyclin D2 (sc-593, Santa Cruz), anti-Cyclin D1 (NBP2-32840, Novus Biologicals), anti-β-actin (4970S, Cell Signaling Technology), anti-β-tubulin (T4026, Sigma), and anti-cleaved caspase-3 (9661L, Cell Signaling Technology). Secondary antibodies conjugated to horseradish-peroxidase were: anti-mouse (715-035-150, Jackson Immuno Research), and anti-rabbit (31460, Pierce, Life Technologies). Blots were developed using Pierce ECL reagents, and chemiluminescence was detected by exposing membranes to GE-Amersham film. Blots were scanned into digital files and exposures were selected for densitometry. Densitometric analysis was done using ImageJ.

### ChIP

Chromatin immunoprecipitation was performed using the SimpleChIP Enzymatic Chromatin IP Kit (Magnetic Beads) from Cell Signaling Technologies. PZp53 cells were cultured to confluency, crosslinked with formaldehyde, and YAP1(D8H1X) XP Rabbit mAb (#14074 Cell Signaling Technology) was used to precipitate DNA bound to the YAP1/TEAD complex. Normal Rabbit IgG (#2729 CST) was used as a negative control, and Histone H3 (D2B12) XP Rabbit mAb (#4620 CST) was used as a positive control for immunoprecipitation. After un-crosslinking and isolation of DNA, quantitative real-time PCR was done. Input 2% samples were also evaluated. Putative TEAD1 binding sites up to 5 kb upstream of the *Hells* gene were identified using the Eukaryotic Promoter Database (https://epd.vital-it.ch/index.php), and tiled primer sets were designed to hybridize to these binding sites using PCR Tiler (http://pcrtiler.alaingervais.org/PCRTiler). Primer pairs were ordered from Eurofins (www.eurofinsgenomics.com), and the sequences are listed in Supplementary Table S1. Immunoprecipitation was done three separate times with multiple samples immunoprecipitated each time, and qRT-PCR was done on each set of samples (IP for YAP1, HistoneH3, and IgG).

### Statistical analysis

Graphs were made using GraphPad Prism 8 (GraphPad Software) and were analyzed using ANOVA and paired two-tailed t-tests unless otherwise noted. For Fig. 1a–c, we used one-way ANOVA with Dunnett’s Multiple Comparison, which compares all columns versus the control column. Data for Fig. 6 was analyzed using multiple t-tests to compare YAP1 to IgG at each putative binding site and then the two-stage linear step-up procedure of Benjamini, Krieger, and Yekutieli was applied to avoid false discovery.

(*)*P* < 0.05; (**)*P* < 0.01; (***)*P* < 0.001; (****)*P* < 0.0001; (no asterisk) not significant.

## Supplementary information

Supplementary information

## Data Availability

For patient data analysis, RNA sequencing data is available via the European Genome-phenome Archive (EGA; https://www.ebi.ac.uk/ega/home) accession number EGAD00001004435. For microarray analysis a total of 187 SHH samples that were subtyped into 53 alpha, 31 beta, 37 gamma, 66 delta were used and analyzed for *HELLS* microarray expression under the accession number GSE85217. Raw microarray sequencing data is also available for this dataset via the Gene Expression Omnibus (GEO) accession number GSE85218; however, the publicly available normalized matrix was used. Information about all other data and reagents described under results will be made freely available upon request.
