# Supplementary material for: Upregulation of the chromatin remodeler HELLS is mediated by YAP1 in Sonic Hedgehog Medulloblastoma
Source: Sci Rep. 2019 Sep 20;9:13611. doi: 10.1038/s41598-019-50088-1 (PMC6754407; doi:10.1038/s41598-019-50088-1)
Supplement: Supplementary file 1 — Supplementary information [file 41598_2019_50088_MOESM1_ESM.pdf]

Upregulation of the chromatin remodeler HELLS is mediated by YAP1 in Sonic Hedgehog Medulloblastoma

Authors: M. Hope Robinson,[1,2] Victor Maximov,[1] Shoeb Lallani,[3] Hamza Farooq, [5,6,7,8] Michael D. Taylor,[5,6,7,8] Renee D. Read,[3,4] Anna Marie Kenney [1,4]

Supplementary Figure S1a. HELLS is upregulated in SHH murine medulloblastoma. Additional western blot illustrating elevated protein levels of Hells in four murine MB compared to adjacent non-tumor CB. Refer to Figure 3.

Supplementary Figure S1b. HELLS is upregulated in SHH murine medulloblastoma Full western blots illustrating elevated protein levels of Hells in four murine MB compared to adjacent non-tumor CB. Refer to Figure 3.

Supplementary Figure S2. HELLS expression and protein levels are slightly decreased with inhibition of GLI1/2 with concurrent increases in cleaved caspase 3. (a) *Hells* mRNA expression in SHH-N or SAG stimulated CGNPs treated with increasing concentrations of the GLI1/2 inhibitor GANT61. n=3 **(b)** HELLS protein levels in GANT61 treated CGNPs. Blot is representative of 3 biological replicates **(c)** *Hells* mRNA expression in cultured murine medulloblastoma cells (MBCs) treated with increasing doses of GANT61; n=4, P=0.04 **(d)** HELLS protein levels in GANT61 treated MBCs.

Supplementary Figure S3. HELLS in PZp53 cells +/- cyclopamine +/- verteporfin. HELLS regulation in the murine cell line PZp53 is consistent with the regulation observed in primary mouse CGNPs and MBCs

Supplementary Figure S4. Full length Western blots for Figures 1b, 1d, 4b, and 4d.

Supplementary Figure S5. Full length Western blots for Figures 4d and 5.

Supplementary Table S1. List of primers used for chromatin immunoprecipitation.

Supplementary Figure S1. HELLS is upregulated in SHH murine medulloblastoma.

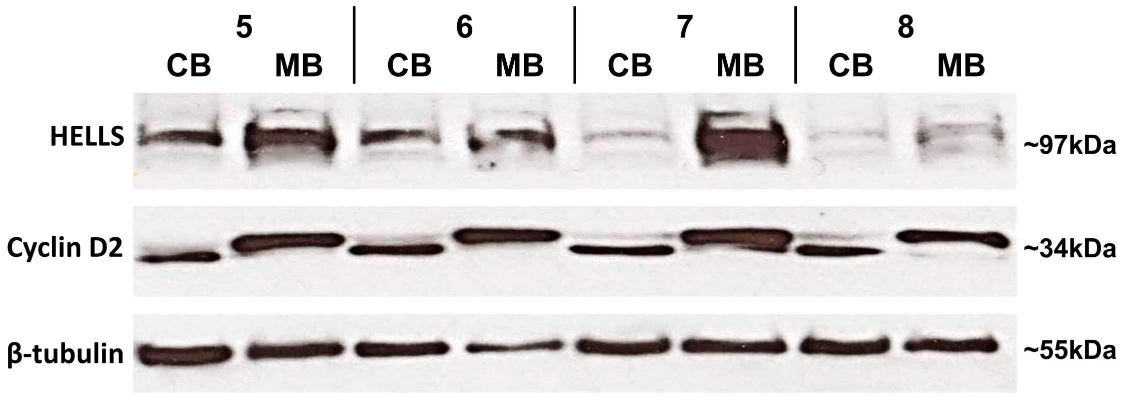

Supplementary Figure S1b. HELLS is upregulated in SHH murine medulloblastoma (Full blots, multiple exposures).

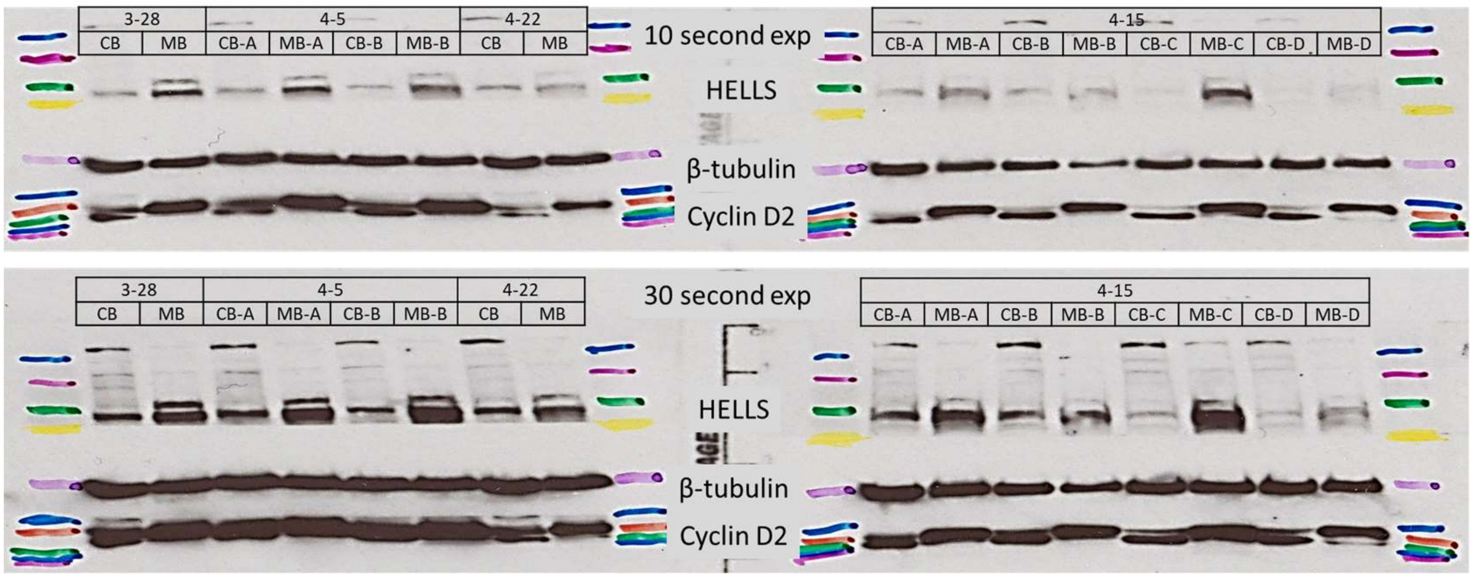

Supplementary Figure S2. HELLS expression and protein levels are slightly decreased with inhibition of GLI1/2 with concurrent increases in cleaved caspase 3.

a.

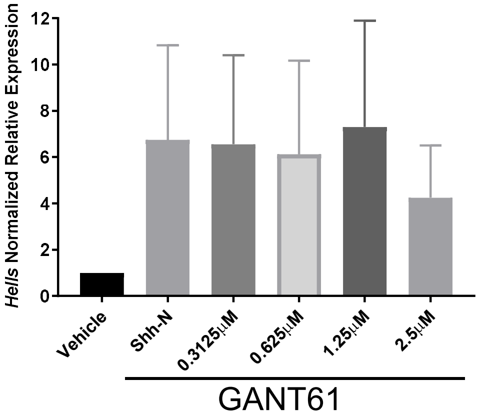

b.

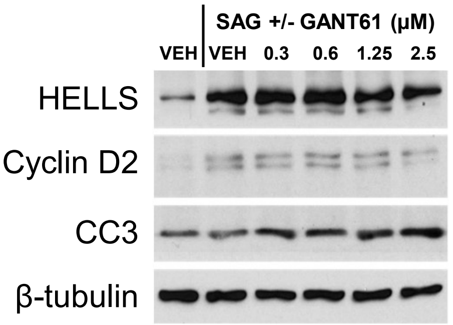

c.

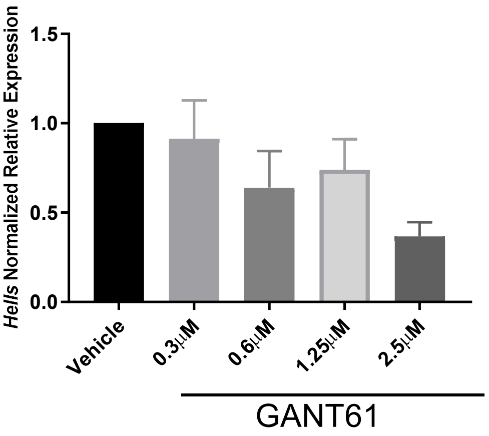

d.

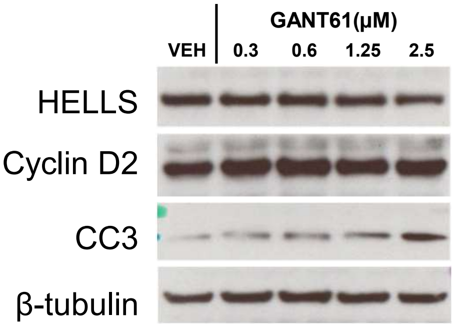

Supplementary Figure S3. HELLS in PZp53 cells +/- cyclopamine +/- verteporfin.

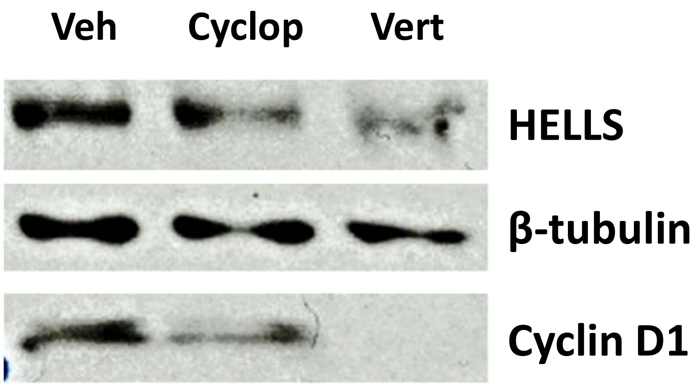

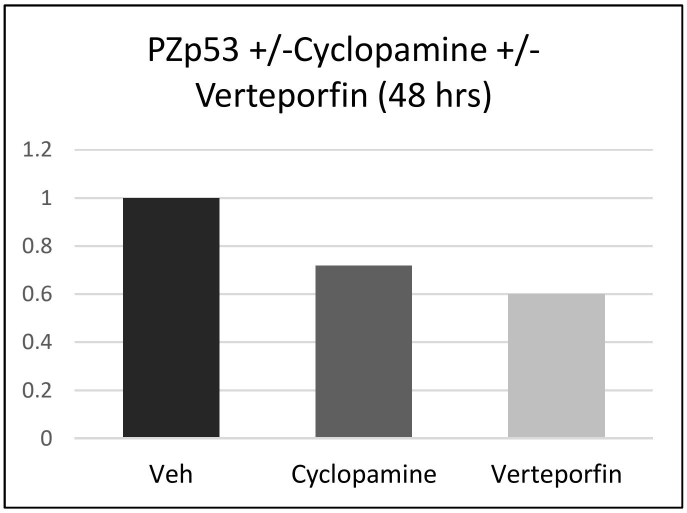

Supplementary Figure S4. Full length Western blots.

Full length blots for Figure 1b. HELLS protein in CGNPs +/- SHH-N +/-Cyclopamine +/- SANT2.

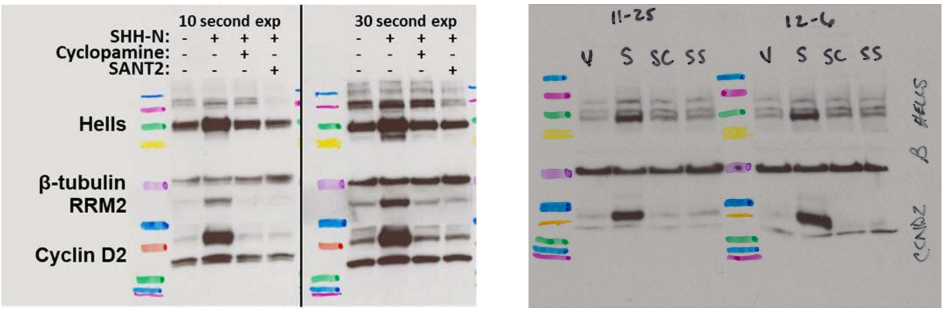

Full blots for Figure 1d. HELLS protein in murine brain development – pooled lysate from >3 separate litters of CD1 mice.

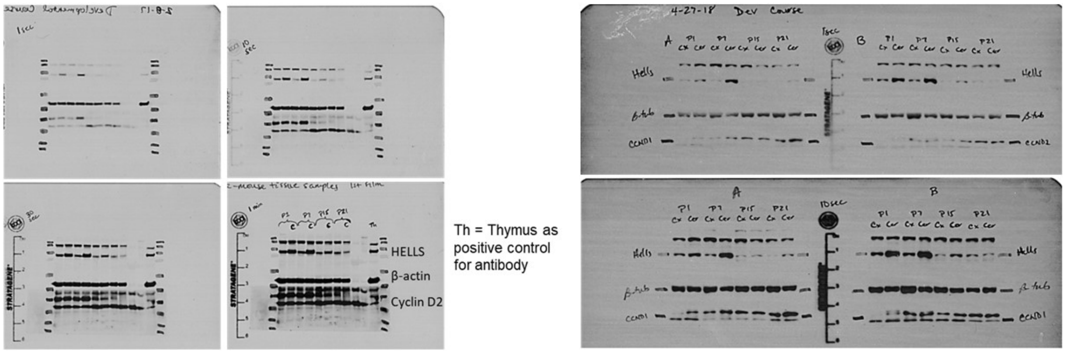

Full blots for Figure 4b. HELLS protein in murine CGNPs +/- SHH-N +/- verteporfin.

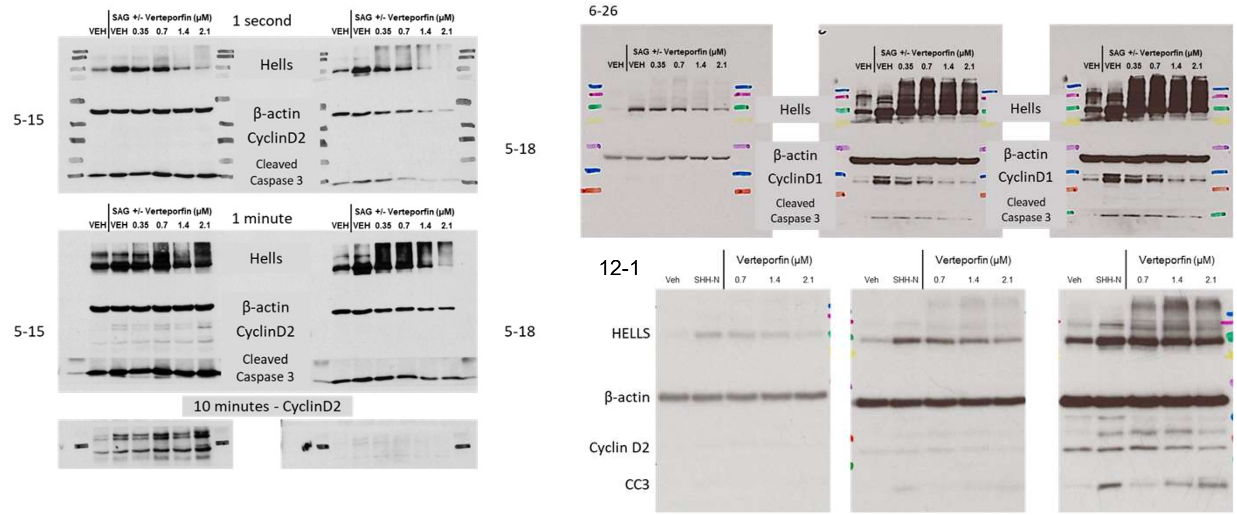

Full blots for Figure 4d. HELLS protein in murine MBC +/- verteporfin.

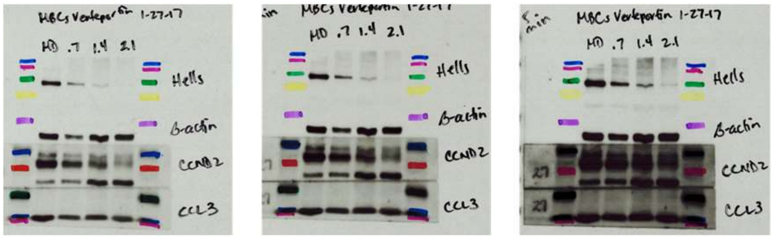

Supplementary Figure S5. Full length Western blots.

Full blots for Figure 4d. HELLS protein in murine MBC +/- verteporfin.

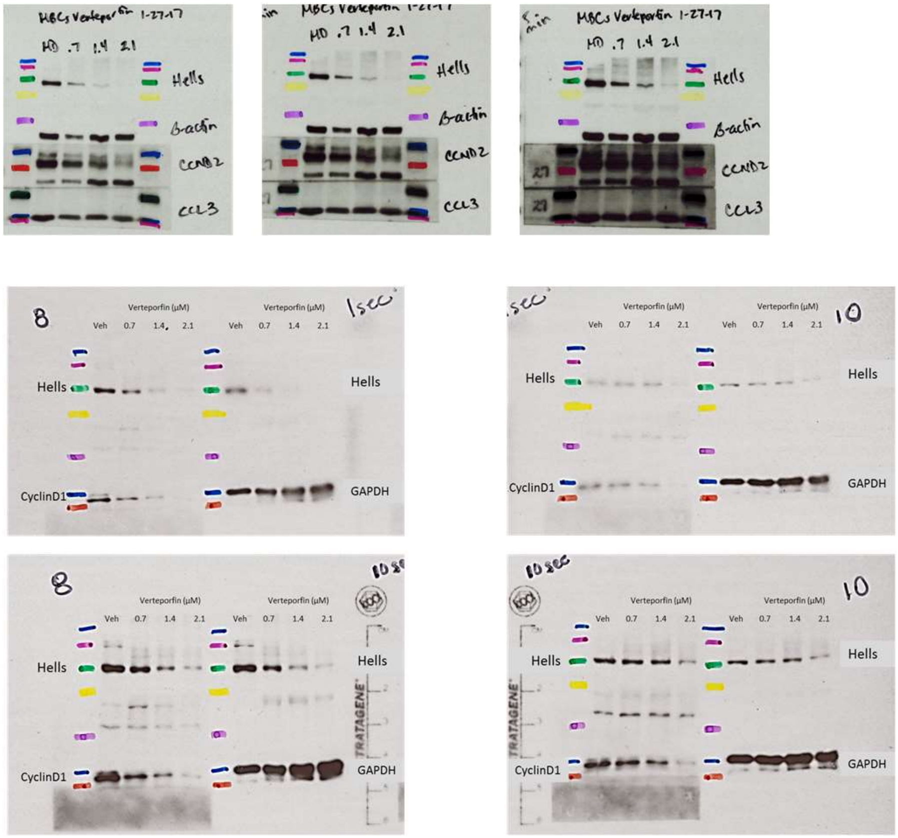

Full blots for Figure 5. HELLS protein levels with YAP1 knockdown.

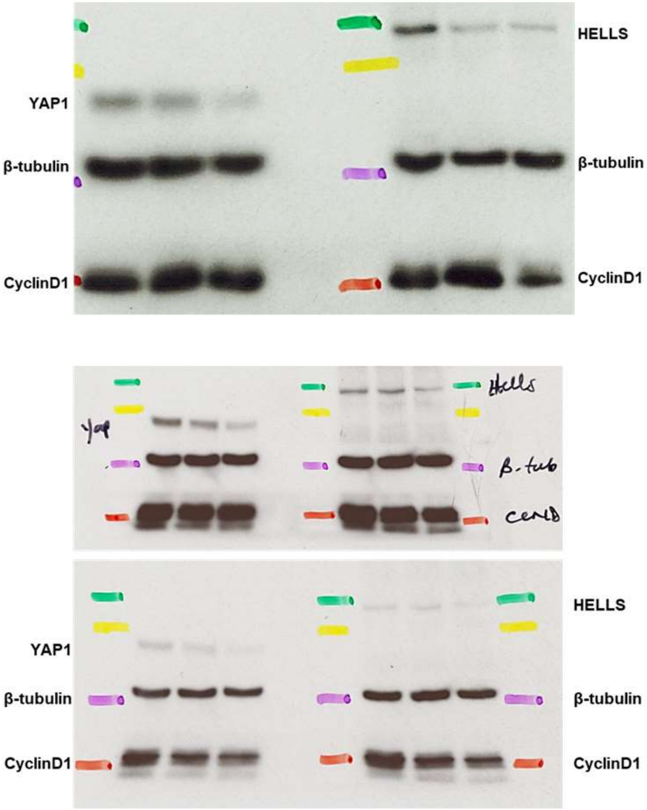

Supplementary Table S1. List of primers used for ChIP.

Name	Sequence	Corresponds to murine *Hells* upstream DNA
Hells-1F	ACCACAGAGCCTGGGACA	-4896
Hells-1R	GGCCAGACACGCATACCC	-4896
Hells-2F	CTGAGATTGGCAACTTGGTGT	-4815
Hells-2R	CCCACTTTCTAGCAGGGACA	-4815
Hells-3F	TTGACCTTTTGTCCCCCTTT	-4699
Hells-3R	AGAGTTTGGAAGCAGTCTGAGC	-4699
Hells-4F	GGCATGCATCACCACACC	-4699, -4310
Hells-4R	CCAGCCTCATCAGCCACA	-4699, -4310
Hells-5F	CACCACACTGGGAGACCTG	-4310,-4264
Hells-5R	CCTTTGGGATGTGTTCTAAGCA	-4310,-4264
Hells-7F	CAGGTCGGCTTCGAACACT	-2391, -2333
Hells-7R	AATGACTTACACTTAGCCAGGCTTT	-2391, -2333
Hells-8F	GGAGAACTGATGTTGCCCAAA	-1958
Hells-8R	CAGGGTTCCTAAATGTCCACTG	-1958
Hells-11F	CAACTCGGGACCATCATTAAATA	-131
Hells-11R	GAGGGATGCGTTAAGCCTTT	-131
Hells-12F	GCGACATTCAAGGCTGGAG	-131
Hells-12R	CGGCTCACGAGATTTGGA	-131
Hells-13F	AGCGCGTCCAAATCTCGT	-131
Hells-13R	AAATTCGCGCGCTTCTCT	-131
